# Racial and ethnic disparities in mortality from gastric and esophageal adenocarcinoma

**DOI:** 10.1002/cam4.3063

**Published:** 2020-06-23

**Authors:** Monika Laszkowska, Angela C. Tramontano, Judith Kim, M. Constanza Camargo, Alfred I. Neugut, Julian A. Abrams, Chin Hur

**Affiliations:** ^1^ Division of Digestive and Liver Diseases Department of Medicine Vagelos College of Physicians & Surgeons Columbia University New York NY USA; ^2^ Institute for Technology Assessment Massachusetts General Hospital Boston MA USA; ^3^ Division of Cancer Epidemiology and Genetics National Cancer Institute Rockville MD USA; ^4^ Department of Medicine Division of Hematology/Oncology Vagelos College of Physicians & Surgeons Columbia University New York NY USA; ^5^ Herbert Irving Comprehensive Cancer Center Vagelos College of Physicians & Surgeons Columbia University New York NY USA

**Keywords:** cardia, esophageal neoplasms, ethnic groups, mortality, race, stomach neoplasms

## Abstract

**Background:**

Racial/ethnic differences in mortality have not been well studied for either non‐cardia gastric cancer (NCGC) or cardia gastric cancer (CGC). The aim of this study was to examine the US mortality rates for these cancer subtypes, as well as esophageal adenocarcinoma (EAC) as a comparator.

**Methods:**

We identified 14 164 individuals who died from NCGC, 5235 from CGC, and 13 982 from EAC in the Surveillance, Epidemiology, and End Results database between 2004 and 2016. Age‐adjusted incidence‐based mortality rates and corresponding annual percent changes (APCs) were calculated. Analyses were stratified by race/ethnicity, age, and stage of disease at diagnosis.

**Results:**

The mortality rate in NCGC was two‐ to threefold higher in blacks, Hispanics, and Asians/Pacific Islanders (PI) than non‐Hispanic whites, and was significant across all age groups and stages of disease (*P* < .01). Mortality in CGC was higher in non‐Hispanic whites than blacks and Asians/PI, particularly in individuals in the 50‐64 year age group and those with stage IV disease. Mortality in EAC was two‐ to sixfold higher in non‐Hispanic whites than all other groups across all age groups and stages of disease. From 2004 to 2016, mortality rates were stable across all racial/ethnic groups in NCGC and CGC, and in minority groups with EAC, but have been rising in non‐Hispanic whites with EAC (APC 3.03, 95% CI 0.17‐5.96).

**Conclusions:**

This is the largest study of incidence‐based mortality in CGC and NCGC and demonstrates racial/ethnic differences in mortality between these subtypes. Mortality rates for NCGC are highest in minority groups, and have been stable in recent years despite declining incidence. Mortality rates for CGC are marginally higher in middle‐aged non‐Hispanic whites with advanced disease, though have remained stable. In contrast, mortality in EAC has been rising for non‐Hispanic whites, in parallel to incidence. Further studies are needed to refine prevention strategies for high‐risk individuals dying from these specific cancer subtypes.

## Graphical abstract

This study examined the US incidence‐based mortality rates for cardia and non‐cardia gastric cancer, as well as esophageal adenocarcinoma as a comparator, and found distinct trends. Mortality was higher in blacks, Hispanics, and Asians/Pacific Islanders compared to non‐Hispanic whites in non‐cardia gastric cancer, but higher in non‐Hispanic whites in cardia gastric cancer and esophageal adenocarcinoma. Further studies are needed to refine prevention strategies for high‐risk individuals dying from these specific cancer subtypes.

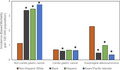

## 1 INTRODUCTION

The two anatomic subtypes of gastric cancer, those of cardia and non‐cardia origin, predominantly impact opposite racial/ethnic groups.^1^, ^2^, ^3^ The incidence of non‐cardia gastric cancer (NCGC) is higher among minority groups (including blacks, Hispanics, Asians/Pacific Islanders, and American Indian/Alaska Natives) than non‐Hispanic whites, while cardia gastric cancer (CGC) has higher incidence in non‐Hispanic whites than other groups.^2^, ^3^, ^4^, ^5^ Trends of CGC have paralleled those of esophageal adenocarcinoma (EAC), suggesting that CGC may be a more similar disease to EAC than to NCGC.^4^, ^5^

While these distinct racial/ethnic incidence patterns in NCGC and CGC are established, few studies have compared differences in survival and mortality trends among racial/ethnic groups in these cancer subtypes. Several studies assessed survival and death certificate mortality in gastric cancer overall, yielding mixed results.^6^, ^7^, ^8^, ^9^, ^10^, ^11^, ^12^, ^13^ Some showed no differences, while others showed higher survival among Asians/Pacific Islanders and inconsistent outcomes in Hispanics.^6^, ^7^, ^8^, ^9^, ^10^

A major limitation in these studies was that they did not stratify outcomes by anatomic subtype for different racial/ethnic groups. Given that CGC and NCGC appear to be distinct disease processes, it is important to understand which subpopulations are at highest risk of dying from each so that interventions can be targeted appropriately. The only study that did investigate this breakdown in both NCGC and CGC utilized the California Cancer Registry and showed no significant differences in survival with respect to race/ethnicity in CGC, but better survival in Asians/Pacific Islanders than non‐Hispanic whites in NCGC.^14^ Two studies looked at outcomes in NCGC alone and also found racial/ethnic differences. Wang et al showed higher survival in Asians than Caucasians, while Schlansky et al found higher death certificate mortality in non‐white races than whites.^15^, ^16^ By comparison, data in EAC have been mixed, but most studies suggest no racial/ethnic differences in survival, similar to CGC.^17^, ^18^, ^19^ These distinctions between trends in CGC and NCGC suggest a need for larger, dedicated studies to examine outcomes in CGC (which may resemble EAC) separately from NCGC.

Incidence‐based mortality is a mortality assessment that allows for the partitioning of data by variables associated with cancer onset, such as age and stage, which is not possible with death certificate mortality alone.^20^ It has proven to be a valuable measure in other types of malignancy.^20^, ^21^, ^22^ Given the critical need to better understand mortality trends between gastric cancer subtypes, the aim of our study was to examine differences in incidence‐based mortality in CGC and NCGC, as well as EAC for comparison, in the US population.

## 2 METHODS

### 2.1 Data source

This study utilized the database of the Surveillance, Epidemiology, and End Results (SEER18) Program of the National Cancer Institute, which includes 18 cancer registry areas (Alaska Native Tumor Registry, Connecticut, Georgia Center for Cancer Statistics (includes 3 registry areas), Greater Bay Area (2 registry areas) and Greater California Cancer Registries, Hawaii, Iowa, Kentucky, Los Angeles, Louisiana, New Mexico, Seattle–Puget Sound, Utah, Detroit, and New Jersey) and covers 28% of the US population.^23^, ^24^ We used the SEER18 incidence‐based mortality database for all analyses.^24^ It should be noted that this database excludes data from Louisiana due to the impact of hurricanes Katrina and Rita in 2005.^25^ The data that support the findings of this study are available from the Surveillance, Epidemiology, and End Results (SEER) Program (www.seer.cancer.gov) SEER*Stat Database (2004‐2016), National Cancer Institute, DCCPS, and Surveillance Research Program, released April 2019, based on the November 2018 submission. Underlying mortality data were provided by NCHS (www.cdc.gov/nchs).24 Given that all SEER data are de‐identified, Institutional Review Board (IRB) approval was not required for this study.

### 2.2 Study population

Data for patients aged 20 years or older who were diagnosed with NCGC, CGC, or EAC from 2004 to 2016 were extracted from the SEER18 database using SEER*Stat (version 8.3.5).^26^ Racial and ethnic classifications of patients were based on those established for the SEER cancer registry, including non‐Hispanic white, Hispanic, black, and Asian or Pacific Islander. Other races/ethnicities were excluded from this analysis. Cancer stage was based on the sixth edition of the AJCC Cancer Staging Manual; patients with unknown stage were excluded. We also excluded cases were diagnosed only by autopsy or death certificate.

CGC patients were defined as those with a primary site of C16.0 (cardia), and NCGC patients were defined as those with a primary site of C16.1‐C16.6 (for fundus, corpus, antrum, pylorus, and lesser and greater curvatures, respectively). EAC patients were defined using International Classification of Diseases for Oncology (ICD‐O‐3) histology codes 8050, 8140‐8147, 8160‐8162, 8180‐8221, 8250‐8507, 8514, 8520‐8551, 8560, 8570‐8574, 8576, and 8940‐8941. Patients with cancers with overlapping tumor locations or cancers for which the tumor location was not specified were excluded.

### 2.3 Statistical analysis

The primary outcome in this study was incidence‐based mortality due to NCGC, CGC, or EAC, which was characterized by age‐adjusted incidence‐based mortality rates. Unlike death certificate mortality, incidence‐based mortality is an assessment that involves tracking cases from diagnosis to death so each mortality event can be linked to characteristics at the time of diagnosis, such as age and cancer stage.^20^, ^21^ Rates were calculated as cancer deaths among cases diagnosed over person‐time at risk and age‐adjusted to the 2000 US standard population. Rates are reported as per 100 000 population. Corresponding annual percent change (APC) in mortality was calculated between 2004 and 2016 using the weighted least squares regression method to assess trends.^27^ The *P*‐value was calculated for each APC using *t* tests to determine whether the trend was significantly different from zero. Mortality analyses were stratified by race/ethnicity, age, and stage of disease at diagnosis (I‐IV). Rates of black, Hispanic, and Asian patients were each compared to non‐Hispanic whites within each age and stage category using the rate ratio method. All statistical analyses were performed using SEER*Stat 8.3.5 software.^26^ Results were statistically significant if *P* < .05.

## 3 RESULTS

A total of 14 164 deaths from NCGC, 5235 from CGC, and 13 982 from EAC were identified in the SEER18 database. Patients with cancers with overlapping tumor locations (n = 3648) or cancers for which tumor location was not specified (n = 6989) were excluded. Patients with unknown disease stage at the time of diagnosis (n = 744 in CGC, n = 2103 in NCGC, and n = 2193 in EAC) were excluded. Among individuals with known stage at diagnosis, non‐Hispanic Native Americans/Alaska Natives, and those with unknown race/ethnicity were excluded due to small sample size (n = 43 for CGC, n = 172 for NCGC, and n = 87 for EAC).

Table 1 shows the number of deaths for each malignancy stratified by sex, age, race/ethnicity, and stage at the time of diagnosis. All cancers had larger proportions of males affected than females, though this was more pronounced in CGC (75.1% male) and EAC (86.7% male) than NCGC (56.1% male). NCGC impacted more individuals aged 20‐49 (11.3%) than CGC (9.7%) or EAC (6.4%), and more individuals over age 65 (65.6%) than CGC (59.3%) and EAC (60.4%). A majority of the cancers in this study were diagnosed at advanced stages, particularly stage IV (58.8% of NCGC, 63.7% of CGC, and 52.6% of EAC).

**Table 1:** Deaths from cardia and non‐cardia gastric adenocarcinoma and esophageal adenocarcinoma according to sex, age, race/ethnicity, and stage at the time of diagnosis

	Non‐cardia gastric cancer	Cardia gastric cancer	Esophageal adenocarcinoma
Total Subjects	14 164	5235	13 982
Sex			
Male	7937 (56.1%)	3980 (75.1%)	12 115 (86.7%)
Female	6227 (43.9%)	1255 (23.9%)	1867 (13.3%)
Age			
20‐49	1546 (11.3%)	508 (9.7%)	888 (6.4%)
50‐64	3280 (23.1%)	1625 (31.0%)	4654 (33.3%)
65+	9338 (65.6%)	3102 (59.3%)	8440 (60.4%)
Race/Ethnicity			
Non‐Hispanic White	6076 (42.9%)	3707 (70.8%)	12 437 (89.0%)
Black	2088 (14.7%)	399 (7.6%)	332 (2.4%)
Hispanic	3323 (23.4%)	469 (8.9%)	957 (6.8%)
Asian/Pacific Islander	2677 (18.9%)	660 (12.6%)	256 (1.8%)
Stage			
I	2227 (15.7%)	782 (15.0%)	1432 (10.2%)
II	1530 (11.0%)	556 (11.0%)	2198 (15.7%)
III	2074 (14.6%)	562 (10.7%)	2991 (21.4%)
IV	8333 (58.8%)	3335 (63.7%)	7361 (52.6%)

**Figure 1:**
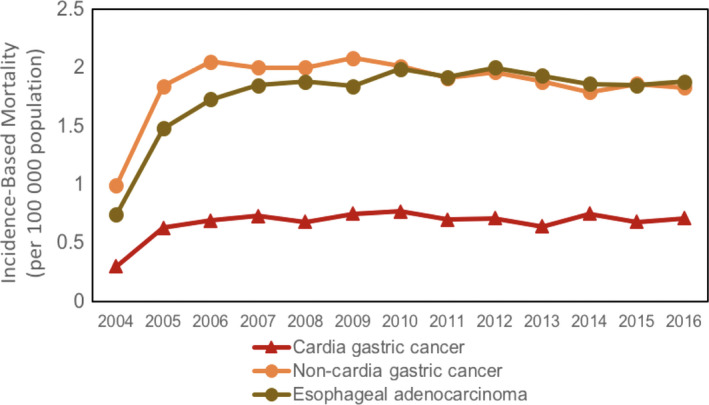
Incidence‐based mortality trends for cardia gastric cancer (APC 1.55, 95% CI 1.22 to 4.40), noncardia gastric cancer (APC 0.49, 95% CI 1.955 to 2.98), and esophageal adenocarcinoma (APC 2.38, 95% CI 0.4s1 to 5.24). APC, Annual Percent Change

The age‐adjusted incidence‐based mortality rate (per 100 000 population) for NCGC was 0.675, for CGC was 1.859, and for EAC was 2.281. Overall mortality rates between 2004 and 2016 were stable for CGC (APC 1.55, 95% CI −1.22‐4.40, *P* = .26) and NCGC (APC 0.49, 95% CI −1.955‐2.98, *P* = .68), but were rising for EAC (APC 2.38, 95% CI 0.41‐5.24). This is illustrated in Figure 1.

**Figure 2:**
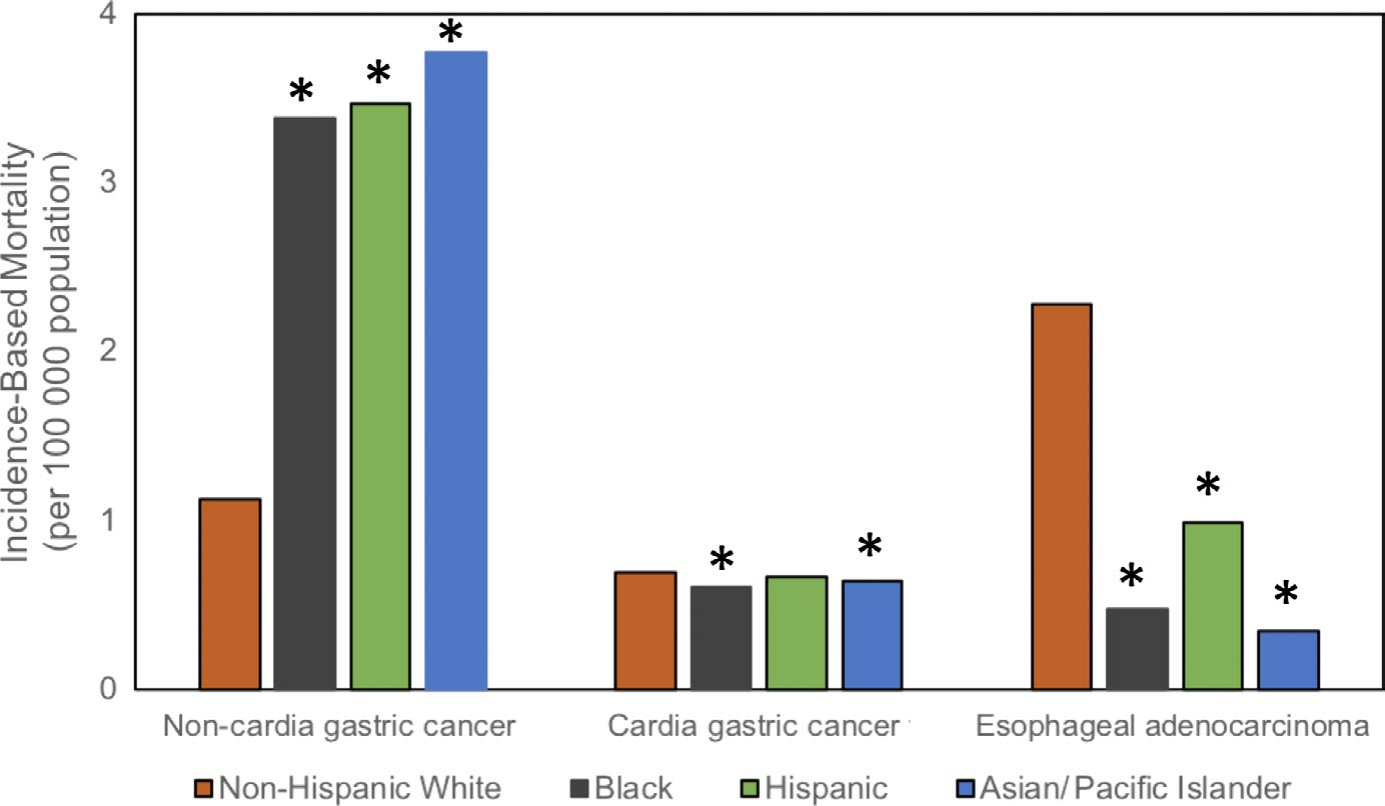
Incidence‐based mortality for non‐cardia and cardia gastric cancer and esophageal adenocarcinoma stratified by race/ethnicity. *P‐value < .01 compared to Non‐Hispanic whites

**Figure 3:**
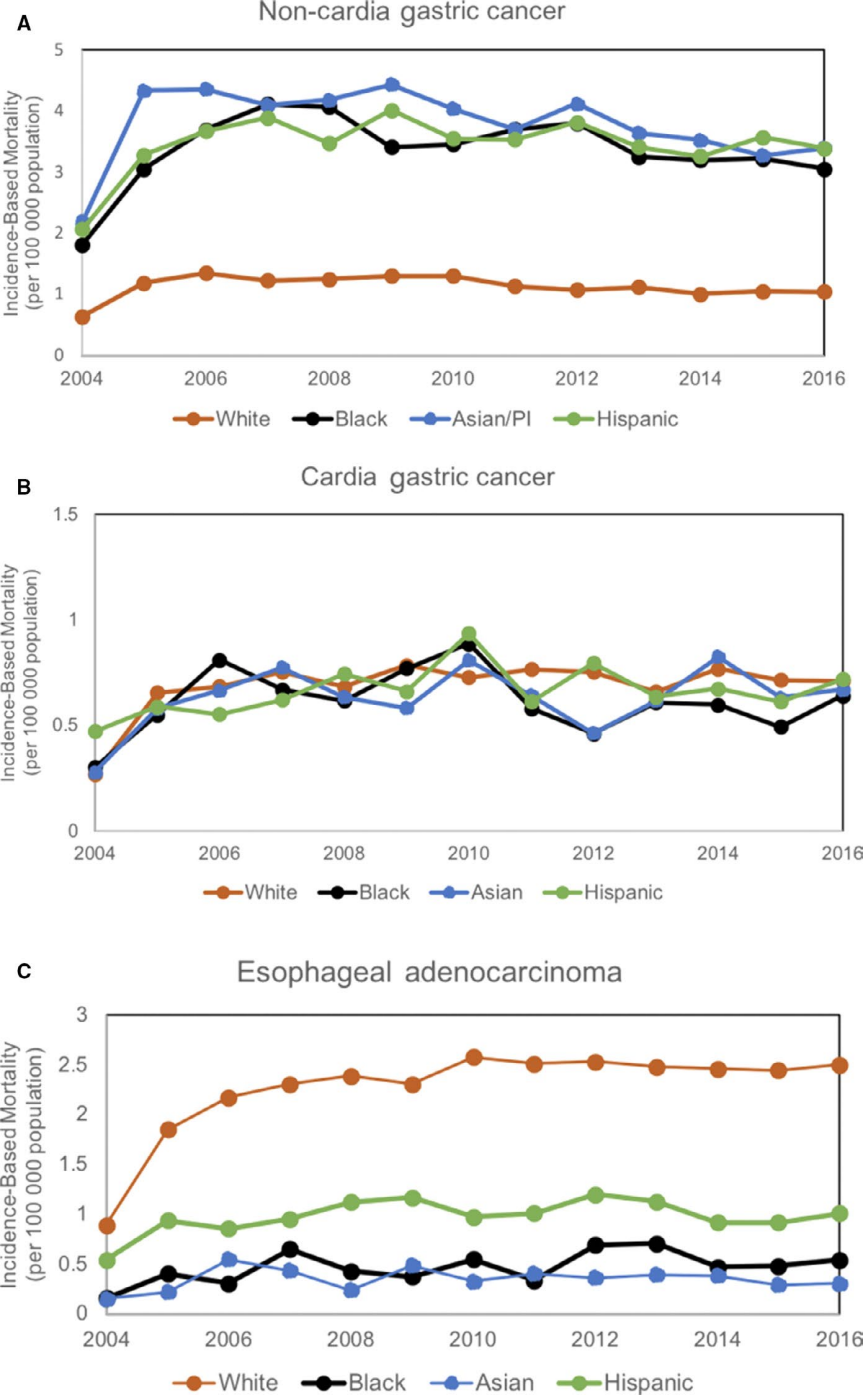
Overall incidence‐based mortality trends for: A, Non‐cardia gastric cancer stratified by race/ethnicity, including non‐Hispanic white (APC 0.75, 95% CI 3.49 to 2.08), black (APC 0.19, 95% CI 3.04 to 2.74), Asian/PI (APC 1.36, 95% CI 3.76 to 1.10), Hispanic (APC 0.38, 95% CI 1.71 to 2.51). B, Cardia gastric cancer stratified by race/ethnicity, including Non‐Hispanic white (APC 1.83, 95% CI 1.23 to 4.99), black (APC 0.73, 95% CI 4.75 to 3.45), Asian/PI (APC 1.42, 95% CI 2.22 to 5.20), Hispanic (APC 1.31, 95% CI 1.49 to 4.20) C, Esophageal adenocarcinoma stratified by race/ethnicity, including Non‐Hispanic white (APC 3.03, 95% CI 0.17 to 5.96), black (APC 3.72, 95% CI 1.60 to 9.34), Asian/PI (APC 0.99, 95% CI 5.90 to 4.18), Hispanic (APC 1.23, 95% CI 1.51 to 4.05). APC, Annual Percent Change; PI, Pacific Islander

Mortality in NCGC was two‐ to threefold higher in blacks (3.38 per 100 000 population), Hispanics (3.47), and Asians/Pacific Islanders (3.77) than in non‐Hispanic whites (1.13). These differences were statistically significant, with *P* < .01 for each group (see Figure 2). Mortality rates by race/ethnicity were further stratified by age and stage of disease at the time of diagnosis in order to account for potential differences in severity of disease. Racial/ethnic differences in mortality rates remained significant across all age groups and stages of disease, with a *P*‐value < .01 in all groups (see Table 2). Corresponding APCs in mortality rates were also calculated. From 2004 to 2016, mortality rates for NCGC did not change significantly when assessed for individual racial and ethnic groups (see Figure 3A).

**Table 2:** Incidence‐based mortality for non‐cardia and cardia gastric cancer and esophageal adenocarcinoma stratified by race/ethnicity, age, and stage of disease at the time of diagnosis between 2004 and 2016

	Incidence‐based mortality (per 100 000)				
	Non‐Hispanic White	Black	Hispanic	Asian/ Pacific Islander	*P*‐value (each group vs Non‐Hispanic White)
Non‐cardia gastric cancer					
Overall	1.13	3.38	3.47	3.77	<.01
By Age (y)					
20‐49	0.15	0.52	0.72	0.52	<.01
50‐64	0.94	2.98	3.25	3.12	<.01
65+	7.73	13.72	13.24	15.77	<.01
By stage					
I	0.18	0.64	0.55	0.6	<.01
II	0.13	0.38	0.38	0.39	<.01
III	0.16	0.48	0.48	0.64	<.01
IV	0.66	1.88	2.06	1.86	<.01
Cardia gastric cancer					
Overall	0.69	0.61*	0.67	0.64*	<.01*
By Age (y)					
20‐49	0.11	0.08	0.14	0.13	NS
50‐64	0.89	0.78	0.72	0.69	<.01
65+	2.45	2.25	2.45	2.36	NS
By stage					
I	0.1	0.11	0.11	0.1	NS
II	0.08	0.1	0.06	0.08	NS
III	0.07	0.07	0.06	0.08	NS
IV	0.44	0.37*	0.43	0.37*	<.01*
Esophageal adenocarcinoma					
Overall	2.28	0.48	0.99	0.35	<.01
By age (y)					
20‐49	0.29	0.06	0.06	0.12	<.01
50‐64	3.11	0.82	1.35	0.4	<.01
65+	8.17	1.51	3.56	1.29	<.01
By stage					
I	0.23	0.05	0.11	0.04	<.01
II	0.36	0.07	0.15	0.04	<.01
III	0.49	0.08	0.19	0.08	<.01
IV	1.19	0.28	0.54	0.19	<.01

* *P* < .01 for black and Asian/Pacific Islander compared with non‐Hispanic white, but not significant for Hispanic vs non‐Hispanic white

Mortality in CGC was slightly lower in blacks (0.61 per 100 000 population) and Asians/PI (0.64 per 100 000 population) than non‐Hispanic whites (0.69 per 100 000 population), and these differences were statistically significant (*P*‐values < .01, see Figure 2). There was no difference between non‐Hispanic whites and Hispanics (0.67 per 100 000 population). When stratified by age, mortality was significantly higher in non‐Hispanic whites aged 50‐64 than other racial/ethnic groups in that age range (see Table 2). When stratified by stage, mortality was higher in whites diagnosed with stage IV disease, than blacks or Asians/PI (see Table 2). From 2004 to 2016, mortality rates have been stable across all racial/ethnic groups with CGC (see Figure 3B).

On the other hand, mortality was 2.3‐ to 6.4‐fold higher in EAC in non‐Hispanic whites than blacks, Hispanics, and Asians/Pacific Islanders (*P* < .01 for each group, see Figure 2). Mortality rates by race/ethnicity were stratified by age and stage of disease at the time of diagnosis, and these differences remained statistically significant across all age groups and stages of disease (*P*‐values < .01, see Table 2). From 2004 to 2016, mortality rates were stable in minorities with EAC, but have been rising in non‐Hispanic whites with EAC (APC 3.03, 95% CI 0.17‐5.96, see Figure 3C).

## 4 DISCUSSION

In this study, we found that overall incidence‐based mortality rates have been stable for CGC and NCGC in recent years, but have been rising for EAC. Mortality was significantly higher in minority groups with NCGC than non‐Hispanic whites. Mortality rates for CGC are marginally higher in non‐Hispanic whites than blacks or Asian/PIs, particularly in middle‐aged individuals with advanced disease. In contrast, mortality in EAC is higher in non‐Hispanic whites than minority groups. Therefore, this study found distinct racial/ethnic mortality patterns among these three disease processes.

This is the first study to demonstrate a difference in mortality between different races/ethnicities in CGC, though this difference is marginal and mainly seen in middle‐aged individuals with advanced disease. One prior study found no significant differences in survival; however, that study utilized the California Cancer Registry and it is possible that survival and mortality trends may differ by region in the US.^14^

While one prior study demonstrated a rising incidence‐based mortality in EAC, ours is the first study to assess this outcome by race/ethnicity.^28^ Notably, results are different than we would expect given observed survival trends in prior studies, which showed no differences in survival by race/ethnicity once adjusted for other factors, such as treatment modality and comorbidities (though non‐whites had lower survival in unadjusted analyses).^17^, ^29^, ^30^, ^31^, ^32^ One prior study showed a trend toward lower survival in whites than minority groups, though these results were not statistically significant.^18^ Differences in study design may explain some of the differences in findings between prior studies and our study. Most prior studies focused on early stage malignancies, generally span an earlier time period than the current study, and some include squamous cell carcinoma of the esophagus along with EAC in their analyses. Furthermore, these studies generally looked at survival rather than incidence‐based mortality. Given the significant rise in incidence‐based mortality in EAC in non‐Hispanic whites in recent years, it is also possible that this group has surpassed other ethnic groups in mortality. A limitation, however, is that our analysis did not adjust for treatment modalities which could have biased results.

In NCGC, we found that incidence‐based mortality was significantly higher in blacks, Hispanics, and Asians/Pacific Islanders than in non‐Hispanic whites, across all age groups and stages of disease. This is in line with the findings of Schlansky et al, who showed higher death certificate mortality in non‐whites than whites in NCGC.^15^

We demonstrate that mortality in NCGC has been stable in recent years, despite the fact that studies show that incidence in this cancer subtype has been declining.^3^ This suggests that in addition to incidence trends, poor outcomes may be contributing to the unfavorable mortality trends that patients with these cancers are facing, which underscore the need for better preventative interventions in high‐risk populations.

The reasons behind these unfavorable mortality trends in gastric cancer in recent years are not clear. Some studies have shown that screening for *H pylori* infection was highest in the US among Asians but low in non‐Asian minorities, including Hispanics and blacks, despite higher rates of NCGC in these groups.^33^ One possibility is that we are underutilizing opportunities to screen the most high‐risk individuals to mitigate relevant risk factors in order to shift diagnosis to earlier stages and improve cancer‐specific mortality. Countries with higher incidence rates of gastric cancer show better survival rates than countries with lower incidence rates, possibly due to the impact of screening.^1^, ^34^, ^35^ Alternatively, given that Koreans treated in Korea for localized gastric adenocarcinoma have lower mortality than Koreans treated in the US, there may be an opportunity to learn from high‐incidence countries to improve treatment outcomes in the US.^35^

Another possibility is that disparities may exist in management practices. Studies have shown that race and geographic region of diagnosis impact treatment recommendations and gastric adenocarcinoma‐specific survival among individuals with resectable tumors.^36^ For example, blacks with resectable gastric tumors are more likely to receive a recommendation against surgery than individuals of other racial groups.^36^ Zhang et al noted significant differences between racial and ethnic groups in the treatment of gastric cancer across stages, with Asians/Pacific Islanders having the highest rate of surgery plus radiation.^10^

Finally, it is possible that there are underlying biological and pathophysiological factors that predispose certain racial and ethnic groups to have more aggressive disease and worse mortality rates, such as minorities in NCGC and non‐Hispanic whites in CGC and EAC. Early findings suggest that differences in molecular subtypes of advanced gastric cancer impact efficacy of various treatment modalities.^37^ Research is needed into whether these molecular profiles might differ between racial/ethnic groups and may account for some of these disparities. Further understanding these disease processes may be crucial to improving outcomes for these groups in the US.

Our study has many strengths. To our knowledge, this is the largest study to compare incidence‐based mortality in gastric cancer stratified by anatomic subtype. Our findings further confirm and extend the concept that these subtypes are distinct disease entities with different patient characteristics and should be considered separately in further studies. Finally, by using incidence‐based mortality rather than death certificate mortality, we are able to adjust our analysis by age and stage of diagnosis, which helped identify distinctions in subgroups among patients with CGC.

Our study also has several limitations. First, we were unable to adjust for certain factors associated with the risk of gastric cancer, such as *H pylori* infection, smoking, and obesity. While the SEER registry includes data from 18 sites across the US, it is possible that sampling may not be completely representative, particularly given evidence of in‐state variability in data for gastric cancer among racial/ethnic groups in other studies.^7^ There is also the risk of classification bias in how patients are assigned to groups within the SEER database, including anatomic subtype and stage. Nonetheless, given the paucity of data on mortality trends for gastric cancer subtypes in the US, we believe this study provides a valuable insight into high‐risk populations that could be the focus of further interventions to improve mortality.

In conclusion, our study found distinct trends in incidence‐based mortality in CGC and NCGC, as well as EAC. Mortality was higher in blacks, Hispanics, and Asians/Pacific Islanders compared to non‐Hispanic whites in NCGC, but higher in non‐Hispanic whites in CGC and EAC. Further research is needed to better understand the underlying mechanisms driving these differences, so that cancer prevention strategies can be targeted to groups who are at highest risk of dying from these specific cancer subtypes.

## CONFLICT OF INTEREST

Monika Laszkowska: Nothing to disclose. Angela C. Tramontano: Nothing to disclose. Judith Kim: Nothing to disclose. M. Constanza Camargo: Nothing to disclose. Alfred I. Neugut: Consulted for Eisai, Otsuka, United Biosource Corp, Hospira, and EHE Intl.

Julian A. Abrams: Nothing to disclose. Chin Hur: Nothing to disclose.

## AUTHOR CONTRIBUTIONS

Conceptualization: ML, ACT, JK, MCC, AIN, JA, CH. Methodology: ML, ACT, JK, MCC, AIN, JA, CH. Formal Analysis: ML, ACT, JK, MCC, AIN, JA, CH. Investigation: ML, ACT, CH. Data Curation: ML, ACT, CH. Writing—Original Draft: ML, ACT, CH. Writing—Review and Editing: ML, ACT, JK, MCC, AIN, JA, CH. Visualization: ML, ACT, CH. Project Administration: CH. Supervision: CH.

## Data Availability

The data that support the findings of this study are openly available from the Surveillance, Epidemiology, and End Results (SEER) Program (www.seer.cancer.gov) SEER*Stat Database (2004‐2016), National Cancer Institute, DCCPS, and Surveillance Research Program, released April 2019, based on the November 2018 submission.

## References

[1] Crew KD , Neugut AI . Epidemiology of gastric cancer. World J Gastroenterol. 2006;12:354‐362. 16489633 10.3748/wjg.v12.i3.354 PMC4066052

[2] Gupta S , Tao LI , Murphy JD , et al. Race/ethnicity‐, socioeconomic status‐, and anatomic subsite‐specific risks for gastric cancer. Gastroenterology. 2019;156(59–62):e54. 10.1053/j.gastro.2018.09.045 PMC6309455 30267713

[3] Yao Q , Qi X , Cheng W , Xie SH . A comprehensive assessment of the racial and ethnic disparities in the incidence of gastric cancer in the United States, 1992–2014. Cancer Res Treat. 2019;51:519‐529. 29921118 10.4143/crt.2018.146 PMC6473285

[4] Kubo A , Corley DA . Marked multi‐ethnic variation of esophageal and gastric cardia carcinomas within the United States. Am J Gastroenterol. 2004;99:582‐588. 15089886 10.1111/j.1572-0241.2004.04131.x

[5] Wu X , Chen VW , Andrews PA , Ruiz B , Correa P . Incidence of esophageal and gastric cancers among Hispanics, non‐Hispanic whites and non‐Hispanic blacks in the United States: subsite and histology differences. Cancer Causes Control. 2007;18:585‐593. 17406989 10.1007/s10552-007-9000-1

[6] Kunz PL , Gubens M , Fisher GA , Ford JM , Lichtensztajn DY , Clarke CA . Long‐term survivors of gastric cancer: a California population‐based study. J Clin Oncol. 2012;30:3507‐3515. 22949151 10.1200/JCO.2011.35.8028

[7] Jim MA , Pinheiro PS , Carreira H , Espey DK , Wiggins CL , Weir HK . Stomach cancer survival in the United States by race and stage (2001–2009): findings from the CONCORD‐2 study. Cancer. 2017;123(Suppl 24):4994‐5013. 29205310 10.1002/cncr.30881 PMC5826592

[8] De B , Rhome R , Jairam V , et al. Gastric adenocarcinoma in young adult patients: patterns of care and survival in the United States. Gastric Cancer. 2018;21:889‐899. 29691758 10.1007/s10120-018-0826-x

[9] Duma N , Sanchez LJ , Castro YS , et al. Gastric adenocarcinoma: clinicopathologic differences among Hispanics and non‐Hispanic whites. A single Institution's experience over 14 years. Ann Gastroenterol. 2016;29:325‐331. 27366033 10.20524/aog.2016.0030 PMC4923818

[10] Zhang G , Zhao X , Li J , et al. Racial disparities in stage‐specific gastric cancer: analysis of results from the Surveillance Epidemiology and End Results (SEER) program database. J Investig Med. 2017;65:991‐998. 10.1136/jim-2017-000413 28442533

[11] Stewart SL , King JB , Thompson TD , Friedman C , Wingo PA . Cancer mortality surveillance–United States, 1990–2000. MMWR Surveill Summ. 2004;53:1‐108. 15179359

[12] Hallowell BD , Endeshaw M , Senkomago V , Razzaghi H , McKenna MT , Saraiya M . Gastric cancer mortality rates among US and foreign‐born persons: United States 2005–2014. Gastric Cancer. 2019;22(5):1081‐1085. 30830640 10.1007/s10120-019-00944-w PMC6697193

[13] Fu WJ . Racial‐sex disparities–a challenging battle against cancer mortality in the USA. J Racial Ethn Health Disparities. 2015;2:158‐166. 26863334 10.1007/s40615-014-0059-6

[14] Klapheke AK , Carvajal‐Carmona LG , Cress RD . Racial/ethnic differences in survival among gastric cancer patients in California. Cancer Causes Control. 2019;30(7):687–696. 31102083 10.1007/s10552-019-01184-0 PMC7172226

[15] Schlansky B , Sonnenberg A . Epidemiology of noncardia gastric adenocarcinoma in the United States. Am J Gastroenterol. 2011;106:1978‐1985. 22008896 10.1038/ajg.2011.213

[16] Wang J , Sun Y , Bertagnolli MM . Comparison of gastric cancer survival between Caucasian and Asian patients treated in the United States: results from the Surveillance Epidemiology and End Results (SEER) database. Ann Surg Oncol. 2015;22:2965‐2971. 25631065 10.1245/s10434-015-4388-4

[17] Tramontano AC , Nipp R , Mercaldo ND , Kong CY , Schrag D , Hur C . Survival disparities by race and ethnicity in early esophageal cancer. Dig Dis Sci. 2018;63:2880‐2888. 30109578 10.1007/s10620-018-5238-6 PMC6738563

[18] Sharaiha RZ , Halazun KJ , Mirza F , et al. Elevated preoperative neutrophil: lymphocyte ratio as a predictor of postoperative disease recurrence in esophageal cancer. Ann Surg Oncol. 2011;18:3362‐3369. 21547702 10.1245/s10434-011-1754-8 PMC3192937

[19] Nassri A , Zhu H , Muftah M , Ramzan Z . Epidemiology and survival of esophageal cancer patients in an american cohort. Cureus. 2018;10:e2507. 29930885 10.7759/cureus.2507 PMC6007500

[20] Merrill RM , Lyon JL . Explaining the difference in prostate cancer mortality rates between white and black men in the United States. Urology. 2000;55:730‐735. 10792091 10.1016/s0090-4295(99)00564-6

[21] Chu KC , Miller BA , Feuer EJ , Hankey BF . A method for partitioning cancer mortality trends by factors associated with diagnosis: an application to female breast cancer. J Clin Epidemiol. 1994;47:1451‐1461. 7730854 10.1016/0895-4356(94)90089-2

[22] Chu KC , Tarone RE , Freeman HP . Trends in prostate cancer mortality among black men and white men in the United States. Cancer. 2003;97:1507‐1516. 12627516 10.1002/cncr.11212

[23] National Cancer Institute . Surveillance, epidemiology, and end results program. About the SEER Registries. https://seer.cancer.gov/registries/. Accessed 20/6/19.

[24] Surveillance, Epidemiology, and End Results (SEER) Program (www.seer.cancer.gov) SEER*Stat Database: Incidence‐Based Mortality ‐ SEER 18 Regs (Excl Louisiana) Research Data, Nov 2018 Sub (2000‐2016) <Katrina/Rita Population Adjustment> ‐ Linked To County Attributes ‐ Total U.S., 1969‐2017 Counties, National Cancer Institute, DCCPS, Surveillance Research Program, released April 2019, based on the November 2018 submission.

[25] Adjustments for Areas Impacted by Hurricanes Katrina and Rita. https://seer.cancer.gov/data/hurricane.html. 2019.

[26] Surveillance, Epidemiology, and End Results (SEER) Program (www.seer.cancer.gov) SEER*Stat Database (2004‐2016), National Cancer Institute, DCCPS, Surveillance Research Program, released April 2019, based on the November 2018 submission.

[27] Walters KA , Li Y , Tiwari RC , Zou Z . A weighted‐least‐squares estimation approach to comparing trends in age‐adjusted cancer rates across overlapping regions. J Data Sci. 2011;8:631‐644. 22375146 PMC3286621

[28] Hur C , Miller M , Kong CY , et al. Trends in esophageal adenocarcinoma incidence and mortality. Cancer. 2013;119:1149‐1158. 23303625 10.1002/cncr.27834 PMC3744155

[29] Tran PN , Taylor TH , Klempner SJ , Zell JA . The impact of gender, race, socioeconomic status, and treatment on outcomes in esophageal cancer: a population‐based analysis. J Carcinog. 2017;16:3. 28974922 10.4103/jcar.JCar_4_17 PMC5615860

[30] Baquet CR , Commiskey P , Mack K , Meltzer S , Mishra SI . Esophageal cancer epidemiology in blacks and whites: racial and gender disparities in incidence, mortality, survival rates and histology. J Natl Med Assoc. 2005;97:1471‐1478. 16334494 PMC2594901

[31] Revels SL , Morris AM , Reddy RM , Akateh C , Wong SL . Racial disparities in esophageal cancer outcomes. Ann Surg Oncol. 2013;20:1136‐1141. 23263780 10.1245/s10434-012-2807-3 PMC4547835

[32] Chen Z , Ren Y , Du XL , et al. Incidence and survival differences in esophageal cancer among ethnic groups in the United States. Oncotarget. 2017;8:47037‐47051. 28410201 10.18632/oncotarget.16694 PMC5564542

[33] Florea A , Brown HE , Harris RB , Oren E . Ethnic disparities in gastric cancer presentation and screening practice in the United States: analysis of 1997–2010 surveillance, epidemiology, and end results‐medicare data. Cancer Epidemiol Biomarkers Prev. 2019;28:659‐665. 30914435 10.1158/1055-9965.EPI-18-0471 PMC10842639

[34] Choi KS , Jun JK , Suh M , et al. Effect of endoscopy screening on stage at gastric cancer diagnosis: results of the National Cancer Screening Programme in Korea. Br J Cancer. 2015;112:608‐612. 25490528 10.1038/bjc.2014.608 PMC4453643

[35] Mueller JL , Kim DH , Stapleton S , et al. Nature versus nurture: the impact of nativity and site of treatment on survival for gastric cancer. Gastric Cancer. 2018. 10.1007/s10120-018-0869-z 30167904

[36] Ulanja MB , Beutler BD , Rishi M , et al. Influence of race and geographic setting on the management of gastric adenocarcinoma. J Surg Oncol. 2019;22(3):446–455. 10.1002/jso.25503 31102468

[37] Kubota Y , Kawazoe A , Sasaki A , et al. The impact of molecular subtype on efficacy of chemotherapy and checkpoint inhibition in advanced gastric cancer. Clin Cancer Res. 2020 10.1158/1078-0432.CCR-20-0075 32156744

